# Supplementary material for: Extracellular stimulation and ephaptic coupling of neurons in a fully coupled finite element-based Extracellular—Membrane—Intracellular (EMI) model
Source: Front Comput Neurosci. 2026 Feb 12;20:1755548. doi: 10.3389/fncom.2026.1755548 (PMC12935970; doi:10.3389/fncom.2026.1755548)
Supplement: Supplementary file 1 [file Data_Sheet_1.pdf]

# Supplementary Information for 'Extracellular Stimulation and Ephaptic Coupling of Neurons in a Fully Coupled Finite Element-Based Extracellular–Membrane–Intracellular (EMI) Model'

Karoline Horgmo Jæger[1] and Aslak Tveito[1]

[1]Simula Research Laboratory, Norway

## S1 Cerebellar Purkinje neuron model parameters

The parameter values for the cerebellar Purkinje neuron are the same as in [1], based on [2]. The expressions for $I_{\text{ion}}$ and $F$ are taken from [2], but intracellular $Ca^{2+}$ dynamics are excluded. The geometry and ion channel conductances of the different parts of the neuron are specified in Tables S1 and S2. In addition, the applied EMI model parameters are specified Table S3.

| | Dendrite ($d < 3.5$ $\mu$m) | Dendrite ($d > 3.5$ $\mu$m) | Dendrite ($d > 8$ $\mu$m) | Soma | AIS | ParaAIS | RN | Collateral |
|---|---|---|---|---|---|---|---|---|
| $l$ | | | | 30 | 17 | 4 | 4 | 100 |
| $d$ | | | | 30 | 1 | 1 | 0.7 | 0.6 |
| Nav1.6 | 0 | 0 | 16 | 214 | 1500 | 0 | 30 | 30 |
| Kv1.1 | 36 | 1.2 | 1.2 | 2 | 0 | 10 | 0 | 0 |
| Kv1.5 | 3.9 | 0.13 | 0.13 | 0 | 0 | 0 | 0 | 0 |
| Kv3.3 | 300 | 10 | 10 | 0 | 0 | 0 | 0 | 0 |
| Kv3.4 | 0 | 0 | 0 | 50 | 10 | 0 | 10 | 20 |
| Kv4.3 | 30 | 1 | 1 | 0 | 0 | 0 | 0 | 0 |
| Kir2.x | 0 | 0.01 | 0.01 | 0.03 | 0 | 0 | 0 | 0 |
| Kca1.1 | 1050 | 35 | 35 | 10 | 0 | 0 | 0 | 0 |
| Kca2.2 | 0 | 1 | 1 | 1 | 0 | 0 | 0 | 0 |
| Kca3.1 | 0 | 2 | 2 | 10 | 0 | 0 | 0 | 0 |
| Cav2.1 | 1 | 1 | 1 | 0.22 | 0.22 | 0 | 0.22 | 0.22 |
| Cav3.1 | 0 | 0.005 | 0.005 | 0.007 | 0.01 | 0 | 0.01 | 0.01 |
| Cav3.2 | 0 | 1.2 | 1.2 | 0.8 | 0 | 0 | 0 | 0 |
| HCN1 | 0.004 | 0.004 | 0.004 | 0.4 | 0 | 0 | 0 | 0 |

**Table S1:** Overview of lengths, $l$, (in $\mu$m) diameters, $d$, (in $\mu$m) and conductances of different types of ion channels (in $mS/cm^2$) in the spatial regions of the Purkinje neuron, based on [2]. There are three Ranvier nodes (RN). Between these, there are myelinated regions of length $l = 100$ $\mu$m and diameter $d = 0.7$ $\mu$m. For the lengths and diameters of the dendric regions, see Table S2.

| Level | 0 | 1 | 2 | 3 | 4 | 5 | 6 | 7 |
|---|---|---|---|---|---|---|---|---|
| $l$ | 15 | 24 | 19.2 | 15.4 | 12.3 | 9.8 | 7.9 | 6.3 |
| $d$ | 10 | 8 | 5 | 3 | 2 | 1.5 | 1 | 1 |

**Table S2:** Lengths, $l$, (in $\mu$m) and diameters, $d$, (in $\mu$m) applied for each branching level in the Purkinje neuron dendrite geometry.

| | Value | Condition | Ref. |
|---|---|---|---|
| $C_m$ | 0.0 $\mu$F/cm$^2$ | Myelinated membrane | [3] |
| | 1.0 $\mu$F/cm$^2$ | Remaining membrane | [4] |
| $\sigma_i$ | 8.2 mS/cm | Purkinje neuron | [2] |
| | 10.0 mS/cm | Pyramidal neuron | [5] |
| $\sigma_e$ | 3.0 mS/cm | Normal, physiological | [6] |
| $\sigma_e/2$ | 1.5 mS/cm | Reduced $\sigma_e$ | Assumed |
| $\sigma_e/5$ | 0.6 mS/cm | Reduced $\sigma_e$ | Assumed |
| $\sigma_e/10$ | 0.3 mS/cm | Reduced $\sigma_e$ | Assumed |
| $\sigma_e/20$ | 0.15 mS/cm | Reduced $\sigma_e$ | Assumed |
| $\sigma_e/50$ | 0.06 mS/cm | Reduced $\sigma_e$ | Assumed |

**Table S3:** EMI model parameters applied in the simulations.

# S2 Neocortical layer 5 pyramidal neuron model parameters

The EMI model representation of a neocortical layer 5 pyramidal neuron model is based the cable model representation in [5], but we have extended the axon modeling applied in [5] to include an axon neck, an axon initial segment (AIS), Ranvier nodes separated by myelinated segments and a collateral. The geometry and membrane model parameters applied in the different neuron segments are specified in Table S4. The geometry is based on [5, 7, 8, 9] and the parameterization is based on the model version from [5] in which the AP is initiated in the axon. The expressions for $I_{\text{ion}}$ and $F$ are taken from [5]. In addition, the applied EMI model parameters are specified Table S3.

| | Apical dendrite | Basal dendrite | Soma | Neck | AIS | RN | Collateral |
|---|---|---|---|---|---|---|---|
| $l$ | 1500 | 200 | 25 | 7 | 31 | 4 | 200 |
| $d$ | 1.5–5.9 | 1.0–1.6 | 25 | 3.4 | 3.5 | 1.7 | 1.3 |
| $\bar{g}_{\text{Nat}}$ | 10.745 | 0 | 249.72 | 487.01 | 9740.25 | 487.01 | 487.01 |
| $\bar{g}_{\text{Nap}}$ | 0 | 0 | 0 | 0.292 | 5.834 | 0.292 | 0.292 |
| $\bar{g}_{\text{Kp}}$ | 0 | 0 | 0 | 188.85 | 188.85 | 188.85 | 188.85 |
| $\bar{g}_{\text{Kt}}$ | 0 | 0 | 338 | 77.274 | 77.274 | 77.274 | 772.74 |
| $\bar{g}_{\text{Kv3.1}}$ | 0.904 | 0 | 0 | 473.8 | 473.8 | 473.8 | 473.8 |
| $\bar{g}_{\text{Ca,HVA}}$ | 0.351 | 0 | 0.644 | 0.222 | 0.222 | 0.222 | 0.222 |
| $\bar{g}_{\text{Ca,LVA}}$ | 70.975 | 0.557 | 0.813 | 0.813 | 0.813 | 0.813 | 0.813 |
| $\bar{g}_{\text{SK}}$ | 0.001 | 0 | 99.65 | 0.047 | 0.047 | 0.047 | 0.047 |
| $\bar{g}_{\text{m}}$ | 0.495 | 0 | 0.008 | 13.322 | 13.322 | 13.322 | 13.322 |
| $\bar{g}_{\text{h}}$ | 0.05 | 0.05 | 0.1 | 0.1 | 0.1 | 0.1 | 0.1 |
| $\bar{g}_{\text{leak}}$ | 0.03 | 0.03 | 0.03 | 0.03 | 0.03 | 0.03 | 0.03 |
| $\gamma$ | 0.000637 | 0.000501 | 0.000509 | 0.000525 | 0.000525 | 0.000525 | 0.000525 |
| $\tau_{\text{decay}}$ | 35.7 | 460.0 | 294.7 | 277.3 | 277.3 | 277.3 | 277.3 |

**Table S4:** Overview of lengths, $l$, (in $\mu$m) diameters, $d$, (in $\mu$m) and parameters of the Hay et al. membrane model [5] in the spatial regions of the pyramidal neuron. The conductances $\bar{g}$ are given in units of mS/cm$^2$, $\tau_{\text{decay}}$ is given in units of ms and, $\gamma$ is unitless. There are three Ranvier nodes (RN). Between these, there are myelinated regions of length $l = 80$ $\mu$m and diameter $d = 1.7$ $\mu$m. Note that the reported value of $\bar{g}_{\text{Ca,HVA}}$ and $\bar{g}_{\text{Ca,LVA}}$ for the apical dendrite correspond to the value in an 200 $\mu$m long so-called "hot" zone located about 750 $\mu$m to the left of the soma. In the remaining parts of the apical dendrites, the value of $\bar{g}_{\text{Ca,HVA}}$ is divided by 10 and the value of $\bar{g}_{\text{Ca,LVA}}$ is divided by 100, following [5].

## S3 Comparison of open-loop (MI+E) and closed-loop (EMI) solutions

As discussed in the paper, open-loop algorithms are most frequently used to compute the membrane potential of neurons and the extracellular potential surrounding them [10, 11, 12]. As carefully explained in [12], the open-loop approach involves first solving the membrane problem and then, post hoc, computing the extracellular potential from the membrane currents obtained in the first step. Here, we denote this approach by MI+E, meaning that the membrane (M) equations are solved together with the intracellular (I) space, and the extracellular (E) solution is subsequently computed.

Our aim is now to compare this open-loop approach (MI+E) with the closed-loop EMI model, in which E, M, and I are solved fully coupled. To this end, we define an open-loop version of the EMI model equations. In this approach, the MI system is given by

$$\begin{aligned} \nabla \cdot \sigma_i \nabla u_i = 0, \text{ in } \Omega_i, \qquad & \frac{\partial v}{\partial t} = \frac{1}{C_m}(I_m - (I_{\text{ion}} + I_s)), \text{ at } \Gamma, \\ I_m = -\mathbf{n}_i \cdot \sigma_i \nabla u_i, \text{ at } \Gamma, \qquad & \frac{ds}{dt} = F(t, v, s), \text{ at } \Gamma \end{aligned} \tag{S1}$$

with the assumption $v = u_i$ at $\Gamma$. After the $u_i$ and $v$ solutions are computed by solving the system (S1), $u_e$ is computed by

$$\nabla \cdot \sigma_e \nabla u_e = 0, \text{ in } \Omega_e, \qquad \mathbf{n}_e \cdot \sigma_e \nabla u_e = I_m, \text{ at } \Gamma, \tag{S2}$$

where $I_m$ is collected from the solution of (S1) and the boundary condition on the outer (non-membrane) part of the extracellular boundary is given by $u_e = 0$. These model definitions allow us to cleanly compare the open-loop and closed-loop approaches in a setup where the open-loop and closed-loop models are as similar as possible, only differing in whether MI is coupled with E or not.

Figure S1 shows the membrane potential and the extracellular potential evaluated at an AIS membrane point for the cerebellar Purkinje model. The solutions are computed for four different values of the extracellular conductivity. The most significant difference between the two approaches appears to be the timing of the spikes. We plot the first and the fifth spikes, and we observe that the difference in spike timing increases with time. Furthermore, we note that the difference between the open-loop (MI+E) solutions and the closed-loop (EMI) solutions increases as the extracellular conductivity decreases. This observation is consistent with the results of [13], where the ephaptic current was shown to increase as $O(1/\sigma_e)$.

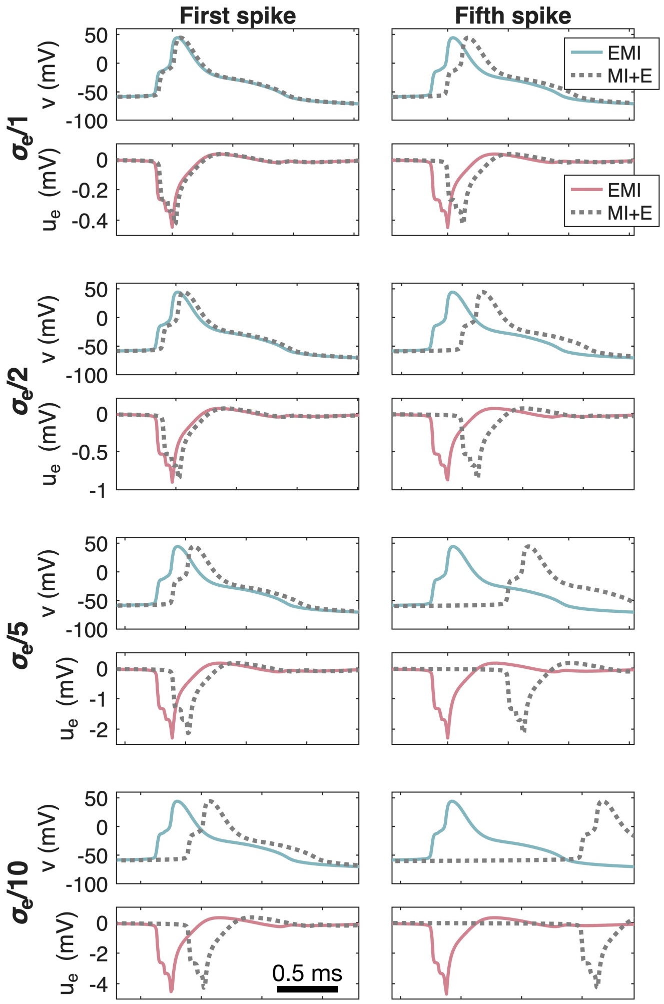


**Figure S1: Comparison between open loop (MI+E) and closed loop (EMI) solutions of a spontaneously firing Purkinje neuron.** We show the membrane potential, $v$, and the extracellular potential, $u_e$, in an AIS membrane point during the first and fifth spikes for four different values of the extracellular conductivity, $\sigma_e$. The default physiological value of $\sigma_e = 3$ mS/cm is divided by 1, 2, 5, or 10 to represent different degrees of assumed $\sigma_e$ reduction. No somatic stimulation is applied.

## References


[1] Karoline H Jaeger and Aslak Tveito. Sometimes extracellular recordings fail for good reasons. *bioRxiv*, pages 2025–07, 2025.

[2] Stefano Masoli, Sergio Solinas, and Egidio D'Angelo. Action potential processing in a detailed purkinje cell model reveals a critical role for axonal compartmentalization. *Frontiers in Cellular Neuroscience*, 9:47, 2015.

[3] AG Richardson, CC McIntyre, and WM Grill. Modelling the effects of electric fields on nerve fibres: influence of the myelin sheath. *Medical and Biological Engineering and Computing*, 38(4):438–446, 2000.

[4] Silvio Weidmann. Electrical constants of trabecular muscle from mammalian heart. *The Journal of Physiology*, 210(4):1041–1054, 1970.

[5] Etay Hay, Sean Hill, Felix Schürmann, Henry Markram, and Idan Segev. Models of neocortical layer 5b pyramidal cells capturing a wide range of dendritic and perisomatic active properties. *PLoS Computational Biology*, 7(7):e1002107, 2011.

[6] Gaute T Einevoll, Christoph Kayser, Nikos K Logothetis, and Stefano Panzeri. Modelling and analysis of local field potentials for studying the function of cortical circuits. *Nature Reviews Neuroscience*, 14(11):770–785, 2013.

[7] Sandrine Romand, Yun Wang, Maria Toledo-Rodriguez, and Henry Markram. Morphological development of thick-tufted layer V pyramidal cells in the rat somatosensory cortex. *Frontiers in Neuroanatomy*, 5:5, 2011.

[8] Patrik Krieger, Christiaan PJ de Kock, and Andreas Frick. Calcium dynamics in basal dendrites of layer 5A and 5B pyramidal neurons is tuned to the cell-type specific physiological action potential discharge. *Frontiers in Cellular Neuroscience*, 11:194, 2017.

[9] Douglas J Bakkum, Marie Engelene J Obien, Milos Radivojevic, David Jäckel, Urs Frey, Hirokazu Takahashi, and Andreas Hierlemann. The axon initial segment is the dominant contributor to the neuron's extracellular electrical potential landscape. *Advanced Biosystems*, 3(2):1800308, 2019.

[10] Wilfrid Rall. Theory of physiological properties of dendrites. *Annals of the New York Academy of Sciences*, 96(4):1071–1092, 1962.

[11] Gary R Holt and Christof Koch. Electrical interactions via the extracellular potential near cell bodies. *Journal of Computational Neuroscience*, 6(2):169–184, 1999.

[12] Aaron R Shifman and John E Lewis. Elfenn: a generalized platform for modeling ephaptic coupling in spiking neuron models. *Frontiers in Neuroinformatics*, 13:35, 2019.

[13] Aslak Tveito, Karoline H Jæger, Glenn T Lines, Łukasz Paszkowski, Joakim Sundnes, Andrew G Edwards, Tuomo Māki-Marttunen, Geir Halnes, and Gaute T Einevoll. An evaluation of the accuracy of classical models for computing the membrane potential and extracellular potential for neurons. *Frontiers in Computational Neuroscience*, 11:27, 2017.
